# Supplementary material for: EndoMAP.v1, a Structural Protein Complex Landscape of Human Endosomes
Source: bioRxiv. 2025 Feb 9:2025.02.07.636106. Preprint. [Version 1] doi: 10.1101/2025.02.07.636106 (PMC11839024; doi:10.1101/2025.02.07.636106)

Figure 3d

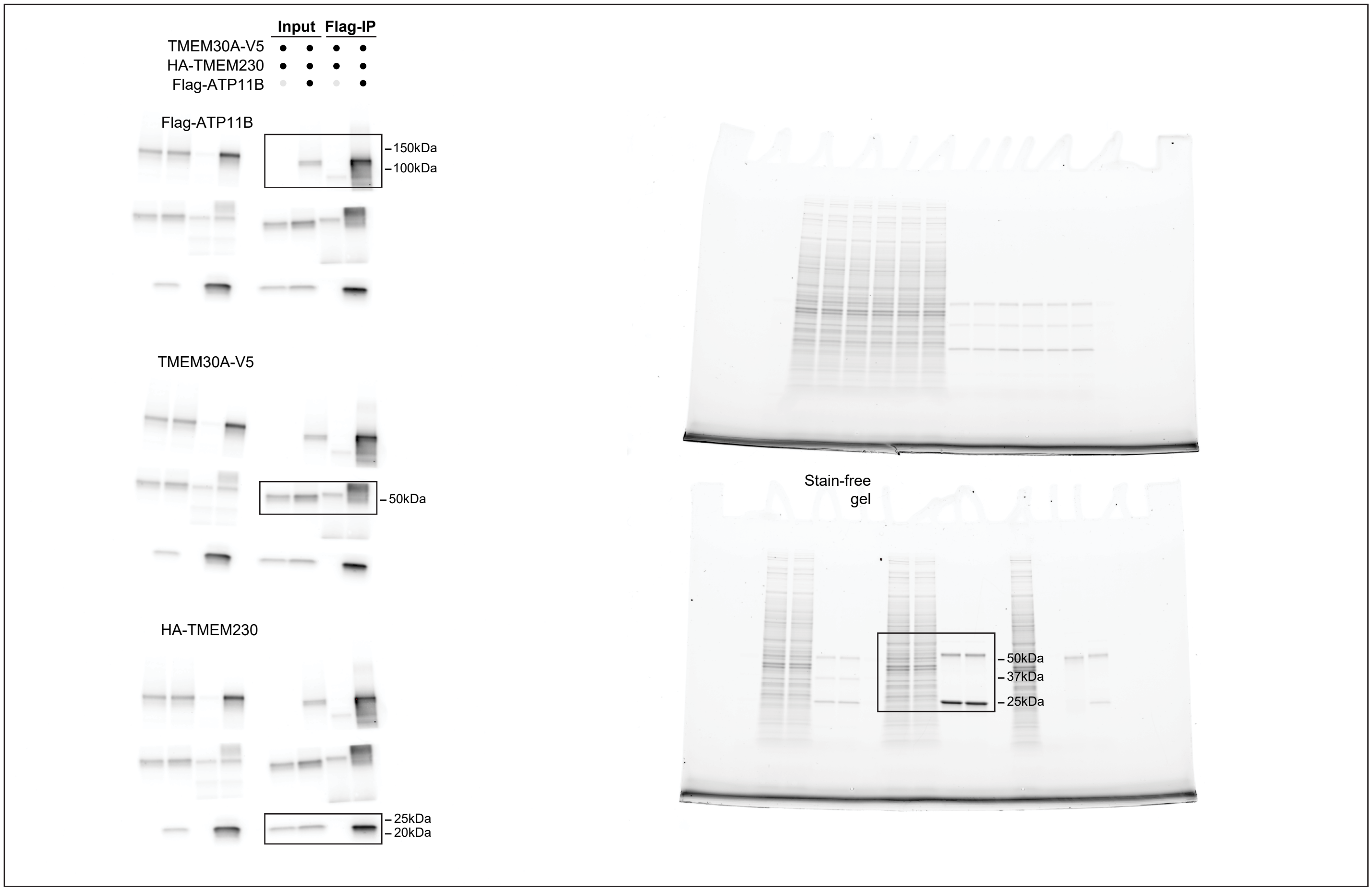

Figure 3h

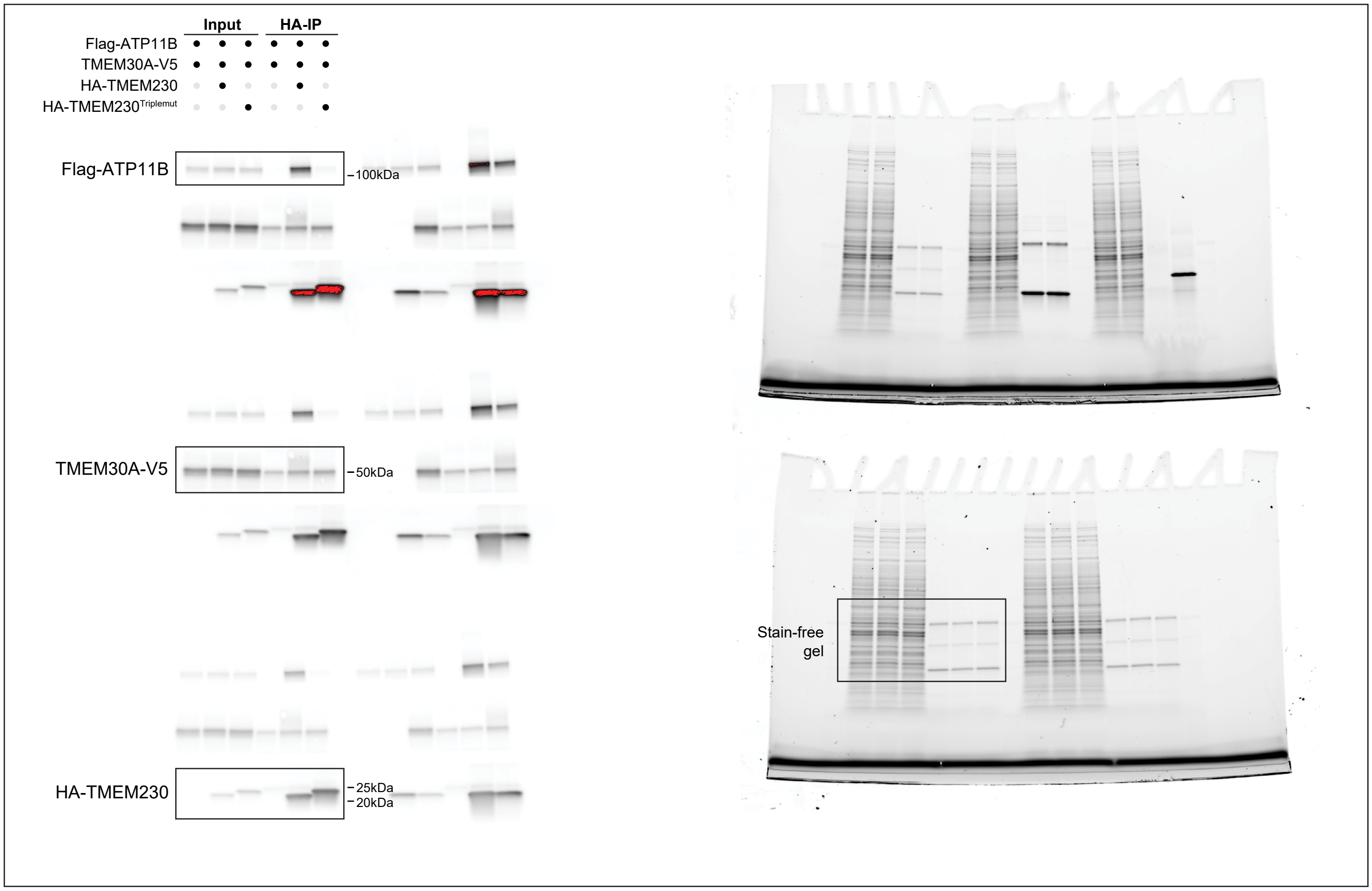

Extended Data Figure 5c

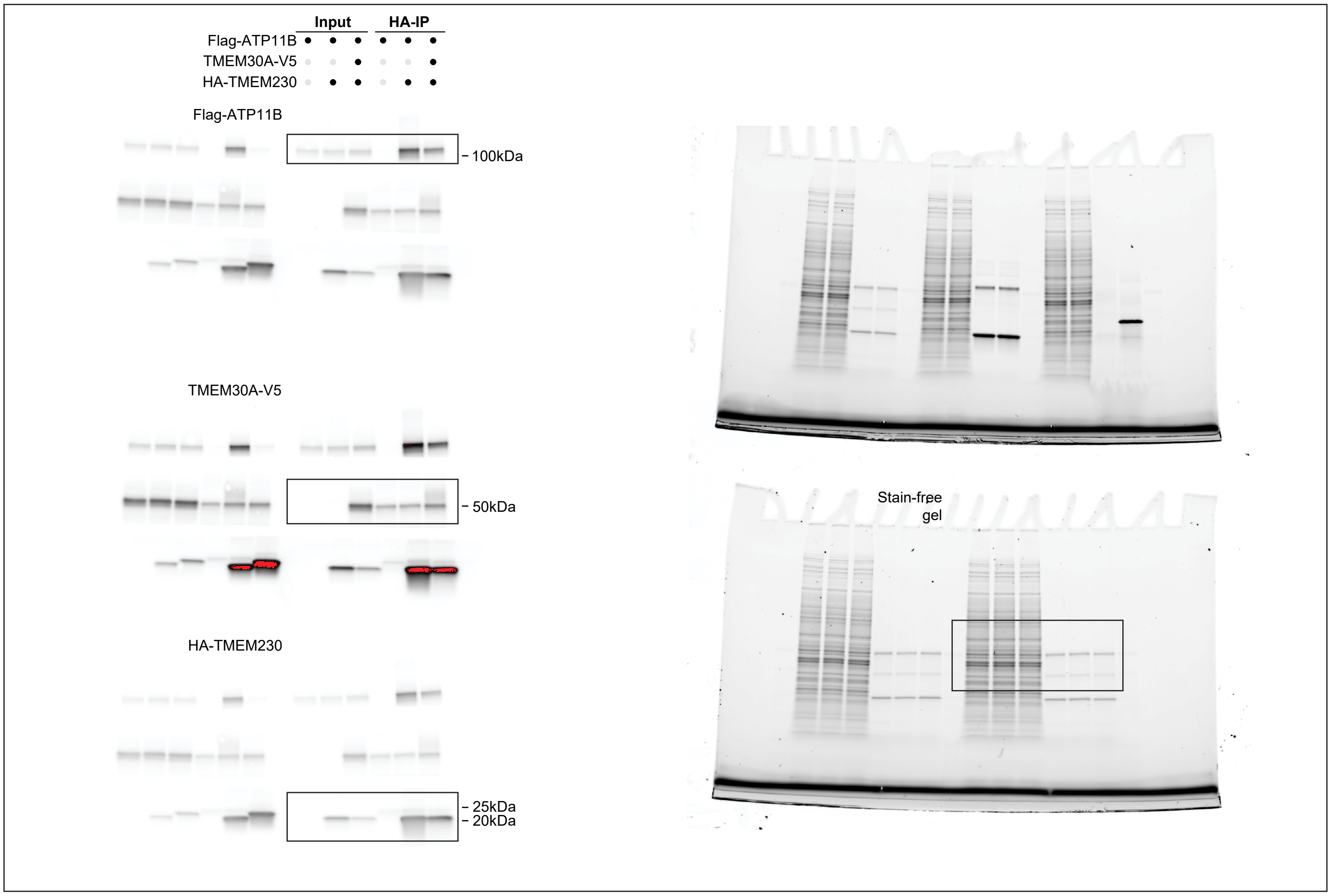

Extended Data Figure 5e

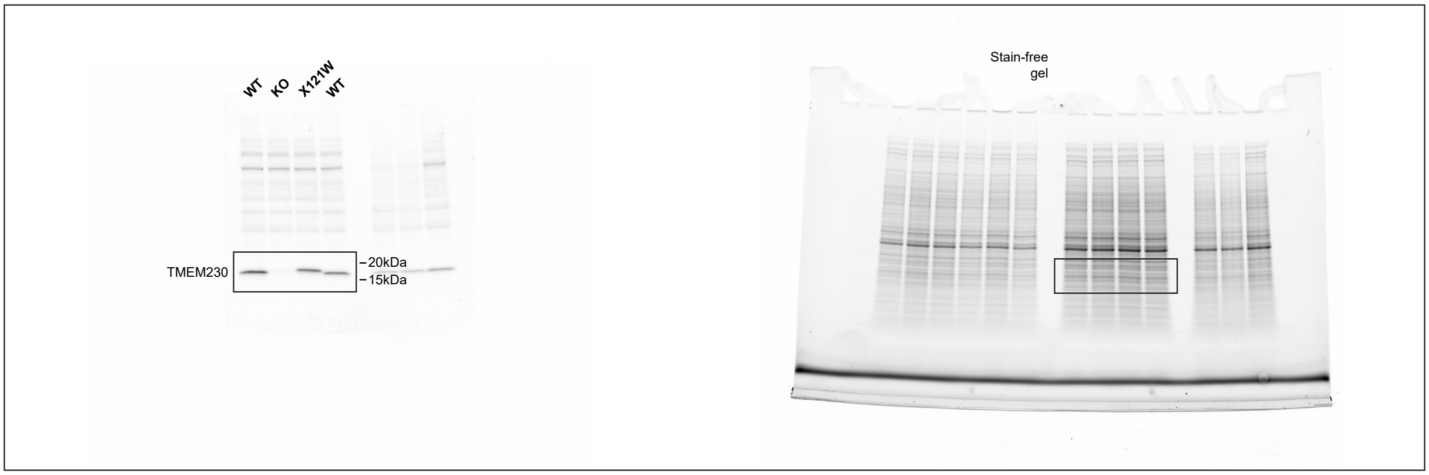

Extended Data Figure 5h

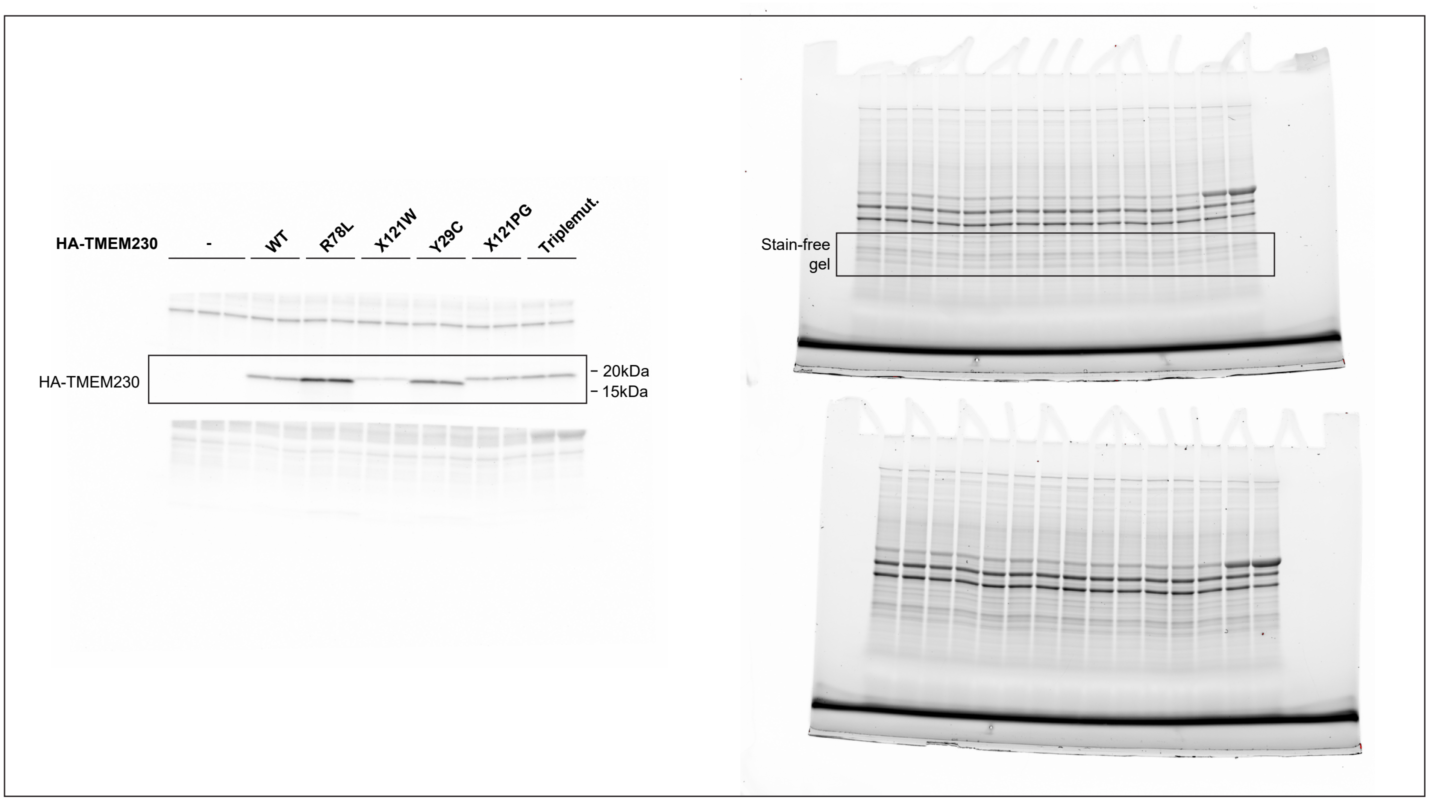

Extended Data Figure 5m

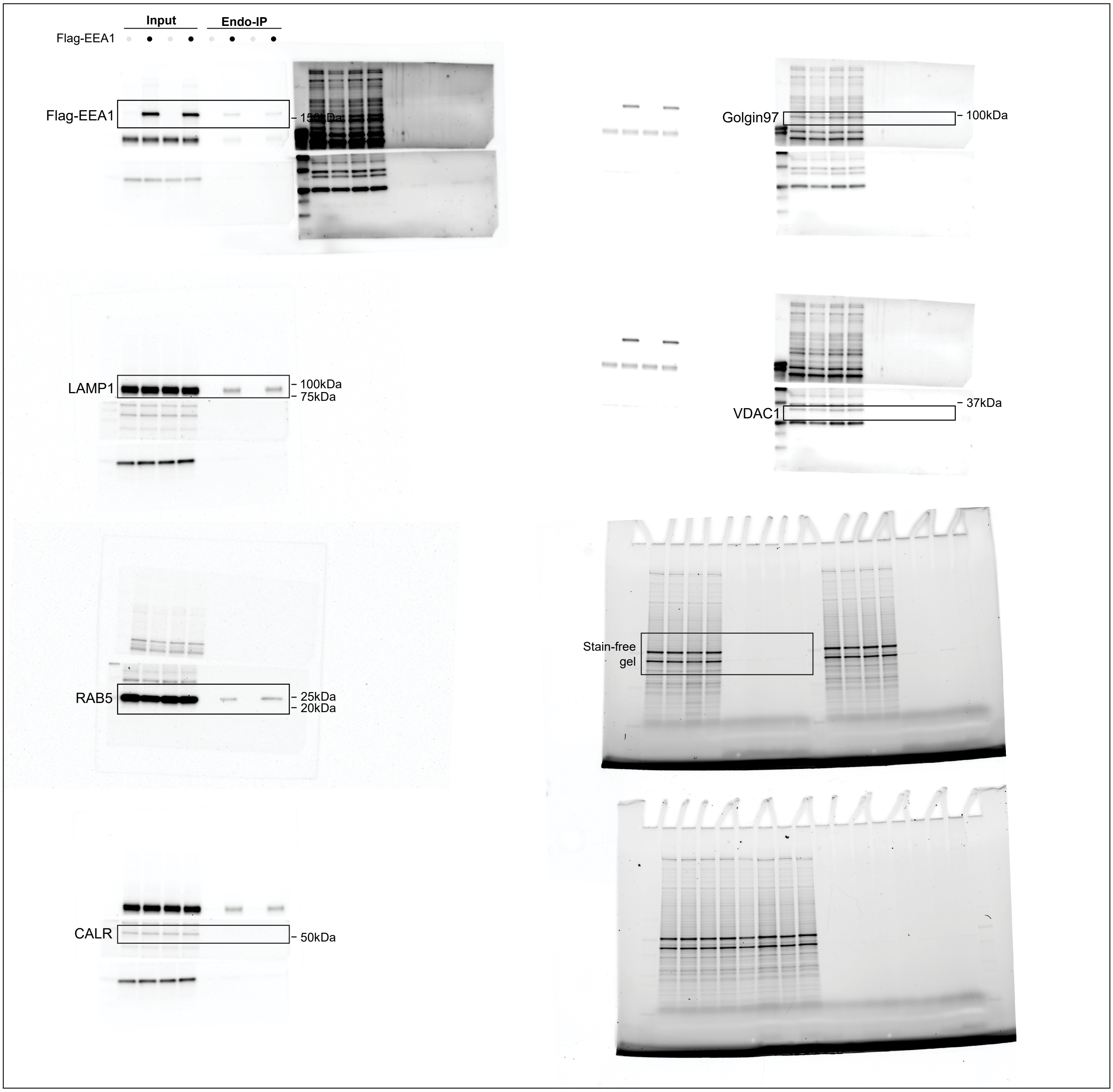

Extended Data Figure 7g

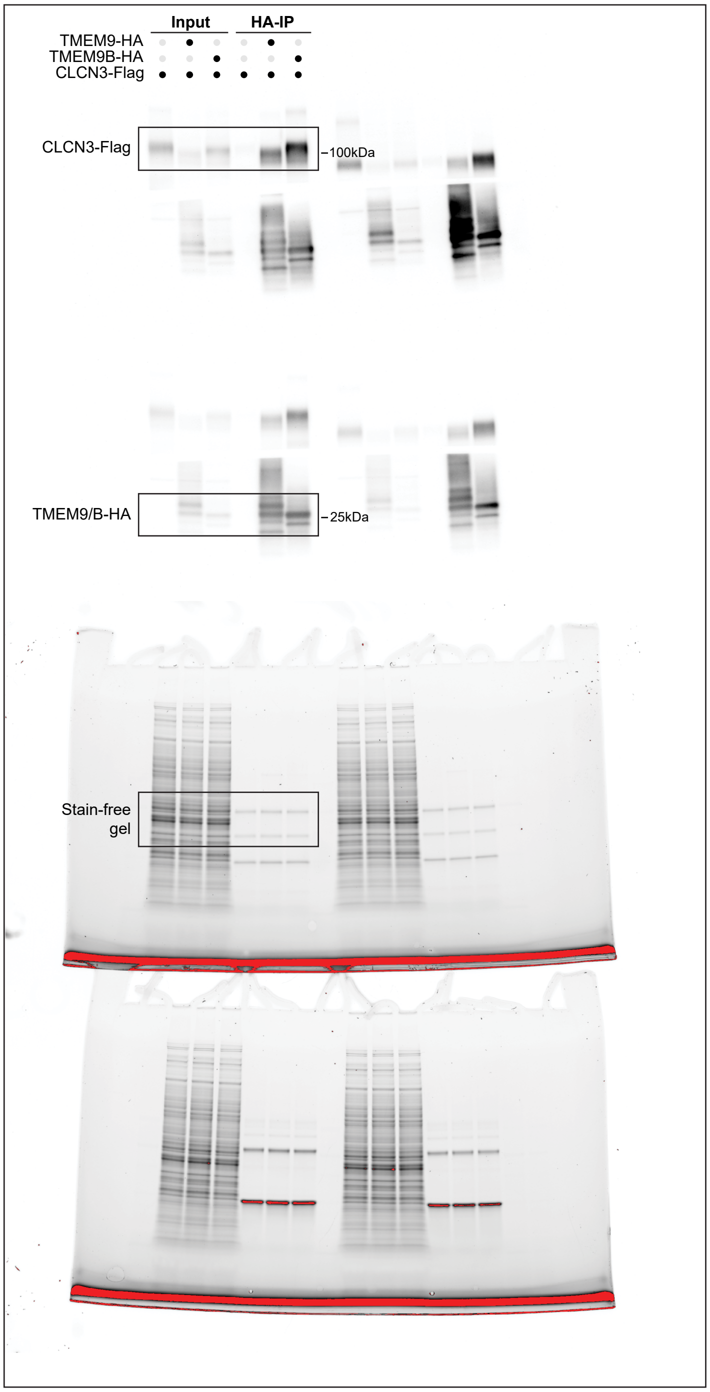

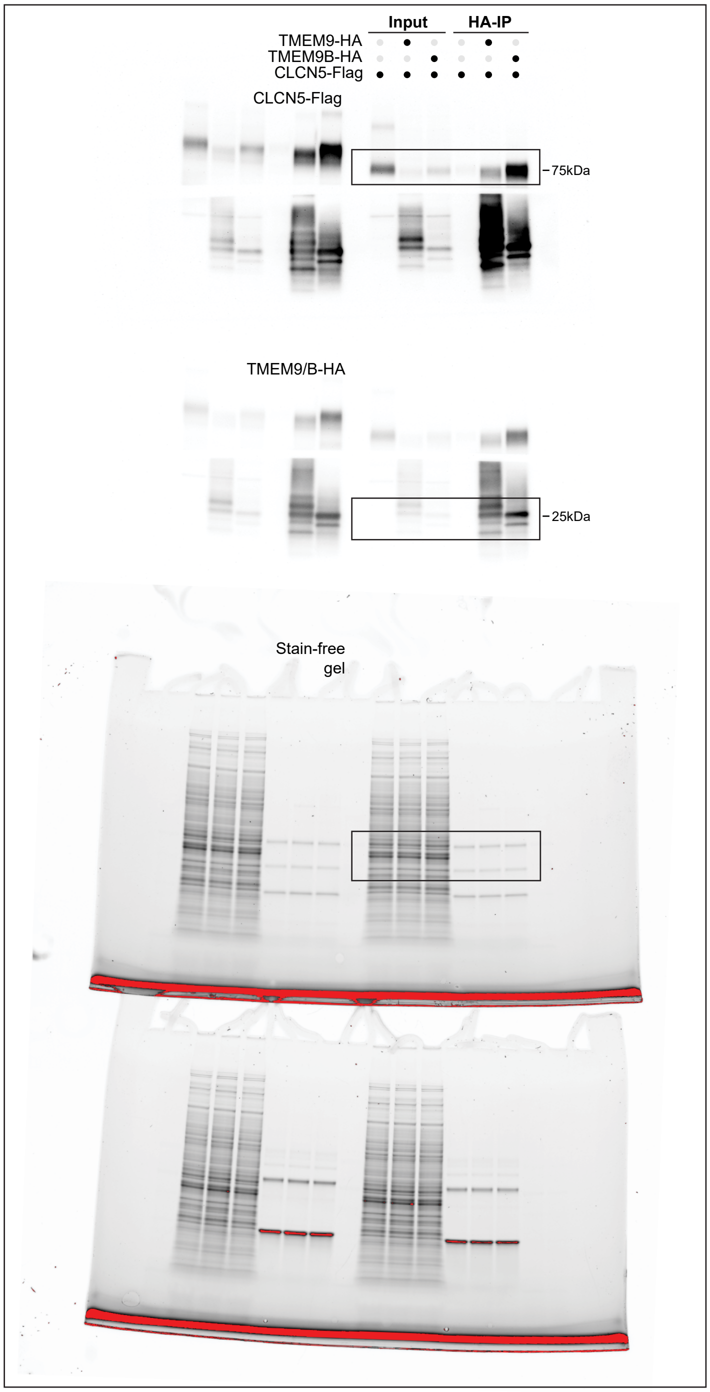

Extended Data Figure 7g (cont.)

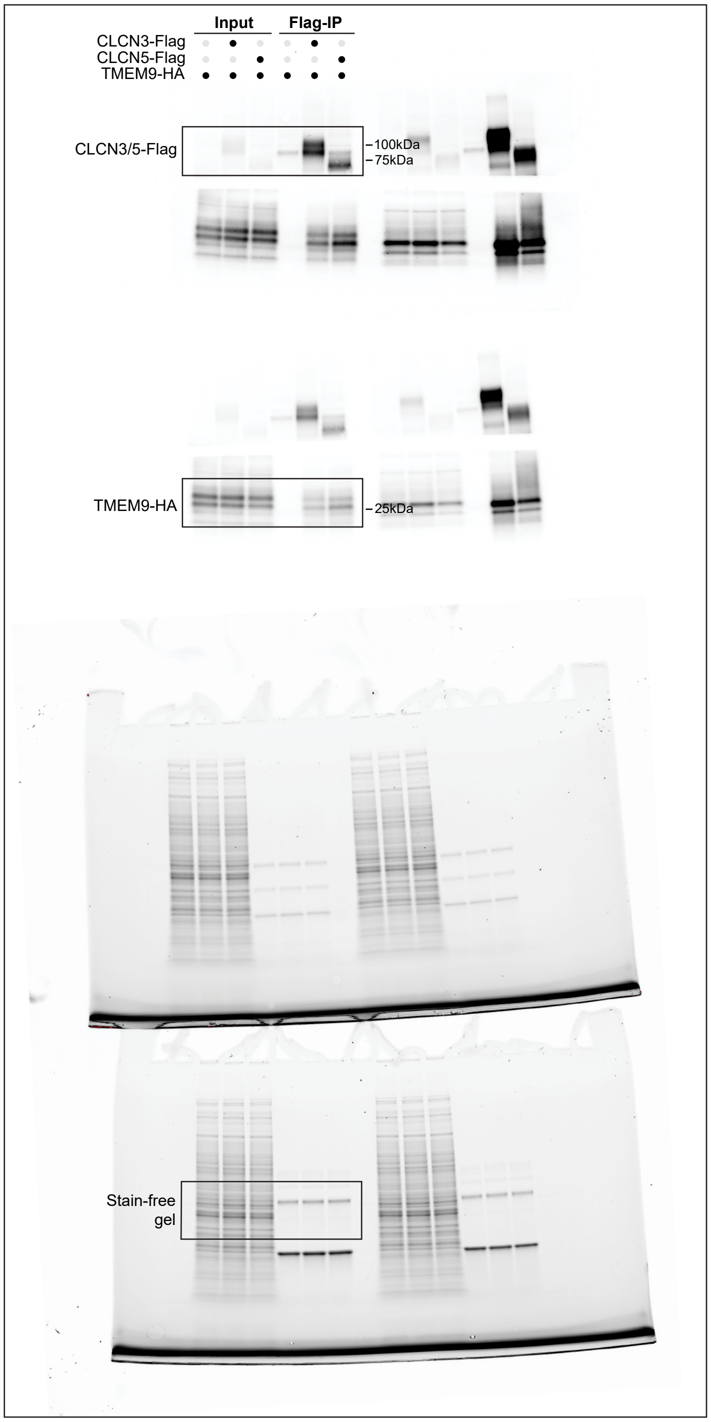

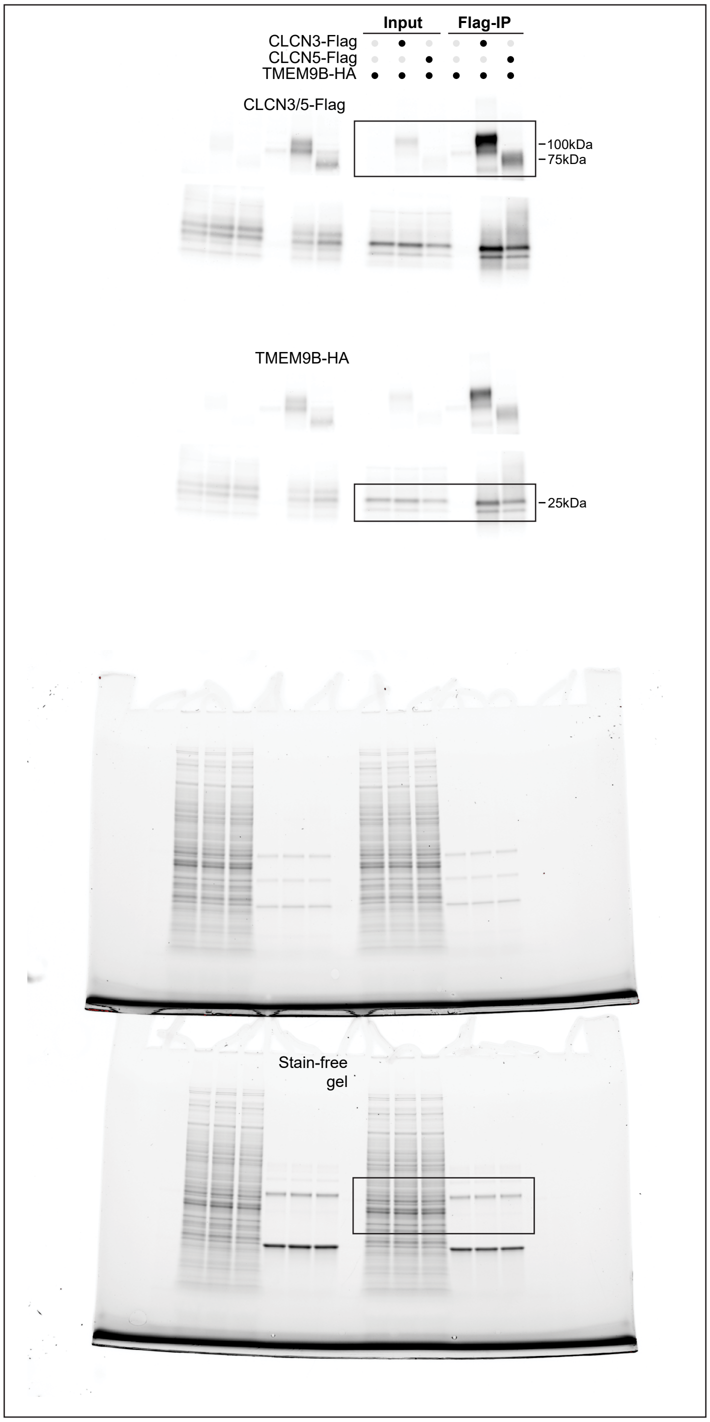

Extended Data Figure 8c

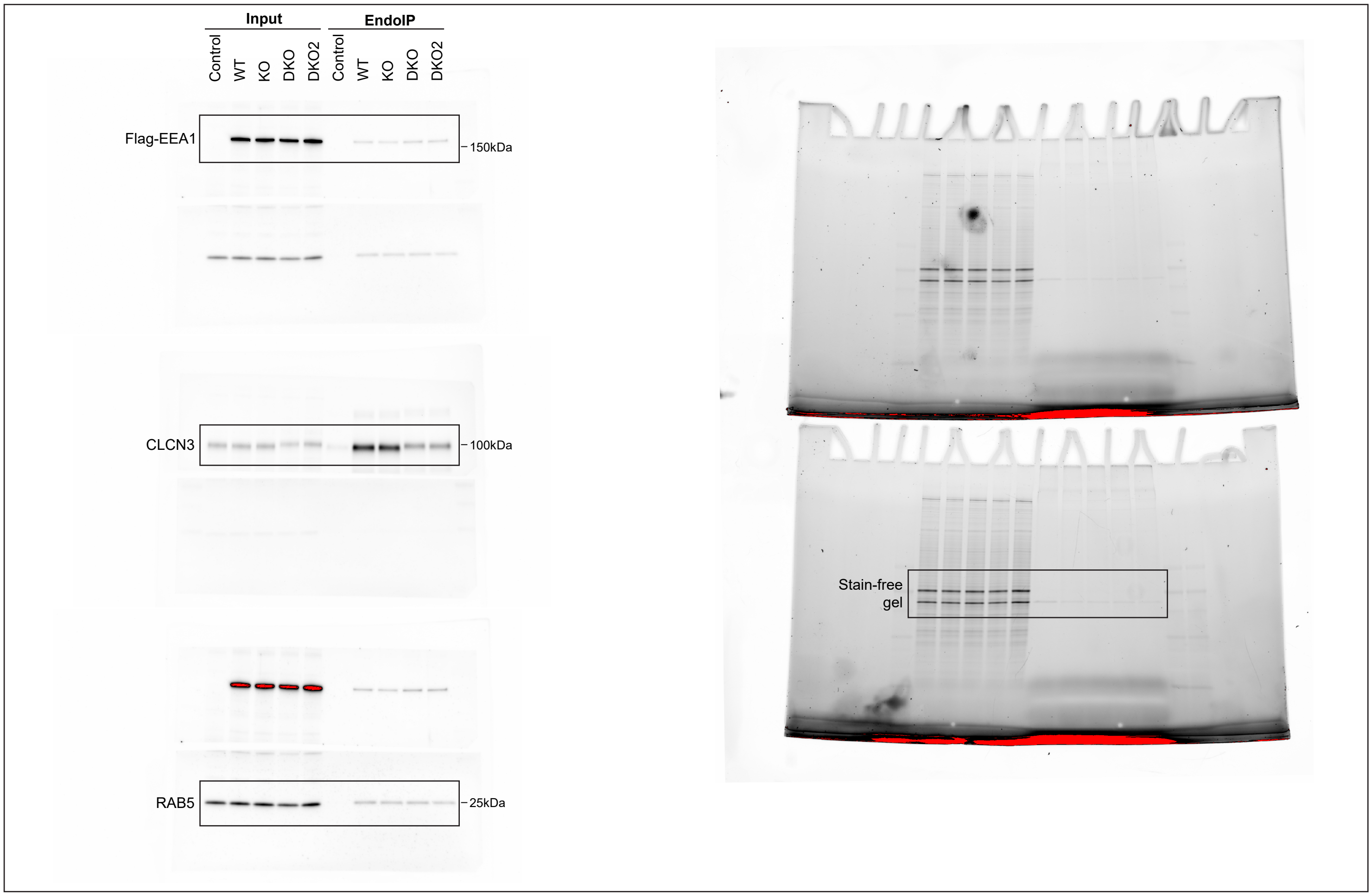

Supplement: Supplement 1 [file media-1.pdf]
