# Supplementary figures and images for: EndoMAP.v1, a Structural Protein Complex Landscape of Human Endosomes
Source: bioRxiv. 2025 Feb 9:2025.02.07.636106. Preprint. [Version 1] doi: 10.1101/2025.02.07.636106 (PMC11839024; doi:10.1101/2025.02.07.636106)

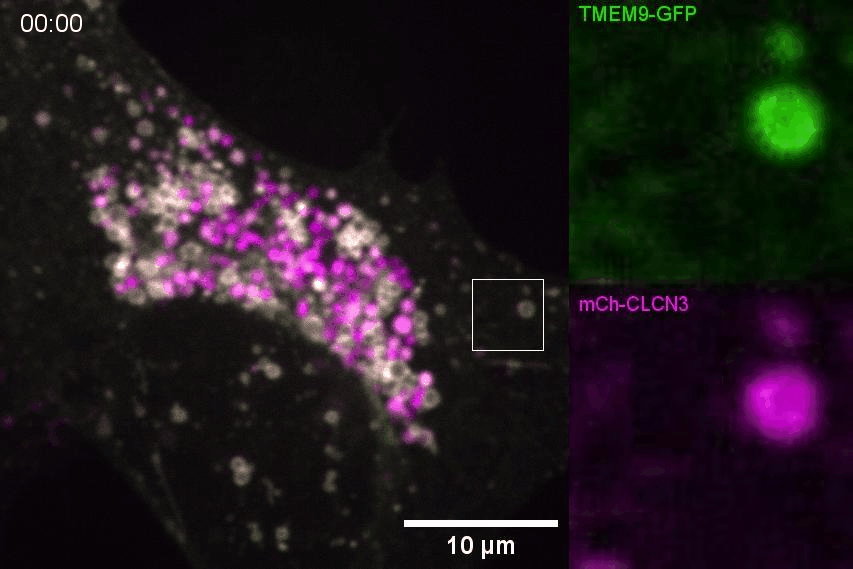

Supplement: Supplement 7 — Supplementary Video 1. Live-cell imaging showing subcellular localization of mCh-CLCN3 and TMEM9-GFP in SUM159 cells (t=2min). [file media-7.gif]
